# Supplementary material for: Splice-Junction-Based Mapping of Alternative Isoforms in the Human Proteome
Source: Cell Rep. Author manuscript; Available in PMC 2020 Jan 15. (PMC6961840; doi:10.1016/j.celrep.2019.11.026)
Supplement: 2 [file NIHMS1546469-supplement-2.zip › DF1/PXD006675/LeftAtrium/LeftAtrium_47_TTN_AESPPPEAIPK.pdf]

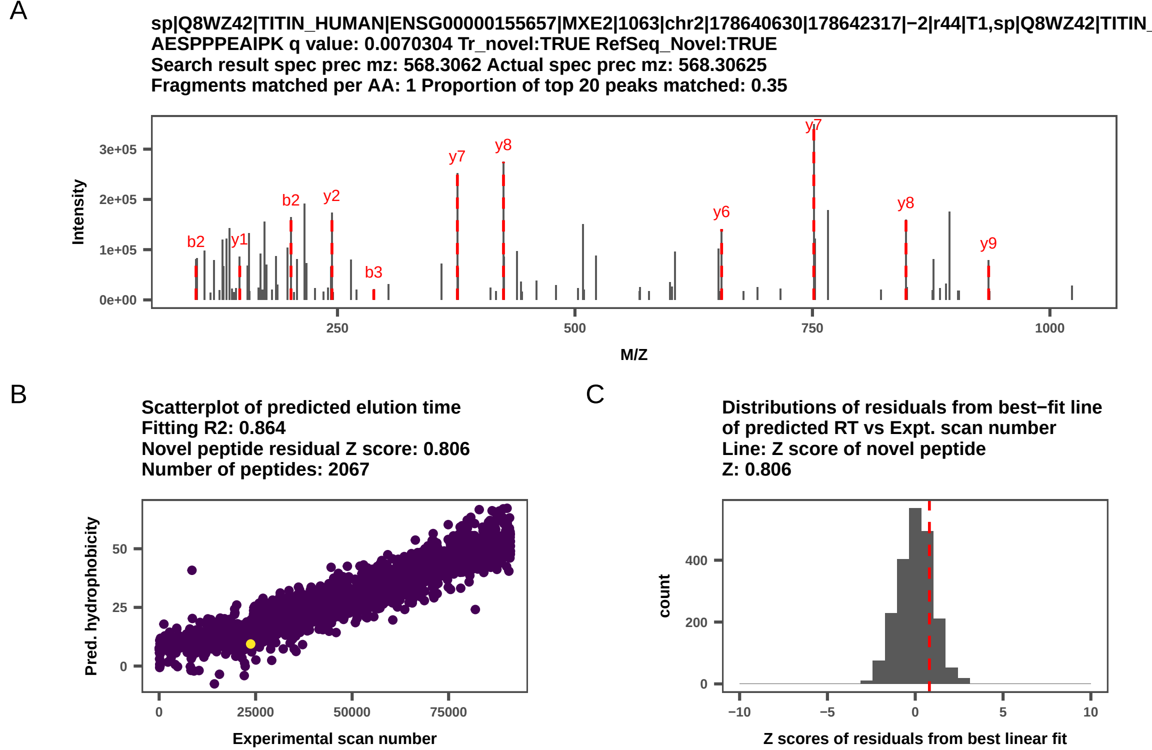
A
sp|Q8WZ42|TITIN_HUMAN|ENSG00000155657|MXE2|1063|chr2|178640630|178642317|−2|r44|T1,sp|Q8WZ42|TITIN_
AESPPPEAIPK q value: 0.0070304 Tr_novel:TRUE RefSeq_Novel:TRUE
Search result spec prec mz: 568.3062 Actual spec prec mz: 568.30625
Fragments matched per AA: 1 Proportion of top 20 peaks matched: 0.35
Intensity
M/Z
b2
y1
b2
y2
b3
y7
y8
y6
y7
y8
y9
B
Scatterplot of predicted elution time
Fitting R2: 0.864
Novel peptide residual Z score: 0.806
Number of peptides: 2067
Pred. hydrophobicity
Experimental scan number
C
Distributions of residuals from best−fit line
of predicted RT vs Expt. scan number
Line: Z score of novel peptide
Z: 0.806
count
Z scores of residuals from best linear fit
